# Histone H4 acetylation by immunohistochemistry and prognosis in newly diagnosed adult acute lymphoblastic leukemia (ALL) patients

**DOI:** 10.1186/1471-2407-10-387

**Published:** 2010-07-21

**Authors:** Anjali S Advani, Sarah E Gibson, Elizabeth Douglas, Tao Jin, Xiaoxian Zhao, Matt Kalaycio, Ed Copelan, Ronald Sobecks, Mikkael Sekeres, Shawnda Sungren, Eric D Hsi

**Affiliations:** 1 Department of Hematologic Oncology and Blood Disorders, Taussig Cancer Center, The Cleveland Clinic, Cleveland, OH, USA; 2 Clinical Pathology, The Cleveland Clinic, Cleveland, OH, USA; 3 Quantitative Health Sciences, The Cleveland Clinic, Cleveland, OH, USA

## Abstract

**Background:**

Histone deacetylase (HDAC) inhibitors are a novel anti-tumor therapy. To determine whether HDAC inhibitors may be useful in the treatment of adult acute lymphoblastic leukemia (ALL), we examined the acetylation of histone H4 by immunohistochemistry in newly diagnosed ALL patients and evaluated the impact of acetylation on complete remission (CR) rate, relapse-free survival (RFS), and overall survival (OS).

**Methods:**

Patients ≥18 years of age and an available diagnostic bone marrow biopsy were evaluated. Cox proportional hazards analysis was used to identify univariate and multivariate correlates of CR, RFS, and OS. The variables histone H4 acetylation (positive or negative), white blood count, cytogenetic (CG) risk group (CALGB criteria), and age were used in multivariate analysis.

**Results:**

On multivariate analysis, histone acetylation was associated with a trend towards an improved OS (for all CG risk groups) (HR = 0.51, p = 0.09). In patients without poor risk CG, there was an impressive association between the presence of histone acetylation and an improved CR rate (OR 3.43, p = 0.035), RFS (HR 0.07, p = 0.005), and OS (HR 0.24, p = 0.007). This association remained statistically significant in multivariate analysis.

**Conclusions:**

These data provide a rationale for the design of novel regimens incorporating HDAC inhibitors in ALL.

## Background

Histones are small basic proteins that complex with DNA to form nucleosomes [1]. Five types occur in humans: histone linker H1 and core histones H2A, H2B, H3, and H4. The core histones are targets for post-translational modification such as acetylation [1]. Histone acetylation is determined by the opposing actions of histone acetyltransferases and histone deacetylases (HDACs) [2-4]. Imbalances in histone acetylation can lead to transcriptional dysregulation of genes involved in cell cycle progression and/or apoptosis by nucleosome remodeling. Increased acetylation of histones H3 and H4 has been associated with transcriptional activation of several genes involved in the suppression of tumor growth [1,5,6]. Histone acetylation and expression of HDACs affect prognosis in a number of cancers. Toh et al [7] demonstrated a favorable prognosis in patients with esophageal squamous cell cancer who demonstrated higher levels of acetylated histone H4. Acetylation correlated inversely with depth of cancer invasion, pathologic stage, and expression of the metastasis-associated-protein-1. Krusche et al [8] demonstrated that expression of HDAC-1 was an independent prognostic marker for patients with breast cancer and correlated with improved disease-free survival.

HDAC inhibitors represent a novel anti-tumor therapy, and are effective against some T-cell lymphoproliferative disorders. Treatment with HDAC inhibitors in cutaneous T-cell lymphoma leads to increased histone acetylation. The cure rate for adults with acute lymphoblastic leukemia (ALL) remains low, and novel treatment strategies are needed. To determine whether HDAC inhibitors may be worthwhile evaluating in the treatment of adult ALL, we examined the acetylation of histone H4 in patients with newly diagnosed ALL and evaluated the impact of acetylation on complete remission (CR) rate, relapse-free survival (RFS), and overall survival (OS). Histone H4 was chosen since we have a well-validated immunhistochemical stain (see our work below in the Kasumi cell lines). In addition, histones H4 and H3, in particular, have been associated with transcriptional activation of several genes involved in the suppression of tumor growth [1,5,6].

## Methods

### Patients

This research was approved by the Cleveland Clinic Institutional Review Board. Between 1996 and 2007, all patients ≥18 years of age with newly diagnosed ALL and an available diagnostic bone marrow biopsy performed at the Cleveland Clinic were evaluated. Cytogenetics were defined according to Cancer and Leukemia Group B (CALGB) criteria [9]. Poor risk cytogenetics included: t(9;22), t(4;11), -7, or +8. The remaining cytogenetic abnormalities were characterized as normal or miscellaneous (any other abnormality).

### Immunohistochemistry

B5-fixed bone marrow core biopsies were reviewed for areas with the highest concentration of blasts. A tissue microarray was constructed using 1 mm tissue cores arrayed in duplicate (Beecher Instruments, Sun Prairie, WI). Immunohistochemistry was performed using automated stainers (Ventana Benchmark, Tucson, AZ), and antibody to acetyl-histone H4 (1:200 dilution; polyclonal; Upstate Biotech, Lake Placid, NY), which has specificity for histone H4 acetylated at lysine residues 5, 8, 12, and 16. Heat-induced epitope retrieval was performed using CC1 solution (Ventana Medical Systems). Five hundred blasts were counted in each case and only strong nuclear staining was classified as positive. Based on the distribution of cell counts, cases were classified as strongly positive if strong nuclear staining occurred in ≥40% of the blasts. The 40% was predetermined as the "definition" of positive before the study was done based on our previous analyses in acute myeloid leukemia demonstrating a natural clustering of data above and below 40% (Gibson SE, et al. USCAP Meeting Poster 151, March 2007). The scoring of each sample was performed in a blinded fashion. Two investigators scored the cases, but each case was scored by one investigator. The level of intensity was not included since the staining was strong in all patients with >40% nuclear staining, and weak in patients with ≥40% nuclear staining. This method of measurement for acetyl-histone H4 was validated in the Kasumi-1 cell line which is derived from a myeloid leukemia with t(8;21) (q22;q22) and has been shown to have decreased levels of histone acetylation.

### Cell Culture

Fresh cultured Kasumi-1 cells were cultured in RPMI 1640 medium with 10% fetal bovine serum at 37 degrees Celsius in a humidified atmosphere containing 5% CO_2_. Cells were collected (1 × 10^6 ^cells/mL) and incubated with or without the HDAC inhibitor sodium butyrate (Sigma, St. Louis, MO) for 22 hours. Cells were then washed once with ice-cold phosphate buffered saline. Cells for each indicated conditions were divided into two groups for preparation of both the lysate and cytospin slide. The cells were lysed by incubating with Laemmli buffer (Bio-Rad, Hercules, CA) for 20 minutes on ice, followed by heating at 95 degrees Celsius.

### Western Blot

The samples were separated by SDS-PAGE in 4-15% polyacrylamide gel and then transferred onto a polyvinylidene membrane (Bio-Rad). Blotting was performed with 3% bovine serum albumin containing 0.1% Tween 20 (PBST) for 4 hours. The membrane was then incubated with antibody to acetyl-histone H4 overnight at 4 degrees Celsius followed by corresponding secondary antibody. Detection was performed using enhanced chemiluminescence reagent (Amersham Pharmacia Biotech, Piscataway, NJ). The membranes were then stripped and reprobed with antibody to B-actin (Sigma). For preparation of cytospin slides, cells of each indicated condition (5 × 10^5 ^cells/mL) were centrifuged to glass slides via a cytofunnel device, followed by fixation in 1% formalin for 15 minutes. Slides were dried at room temperature and immunohistochemical staining for acetyl-histone H4 was performed with the same method as used for paraffin-embedded bone marrow samples.

### Statistics

Cox proportional hazards analysis was used to identify univariate and multivariate correlates of CR, OS, and relapse-free survival (RFS). CR was defined as less than 5% bone marrow blasts, absence of extramedullary leukemia, and recovery of peripheral blood counts (neutrophil count >1,000/μL and platelet count >100,000/μL). The following variables were used in univariate and multivariate analysis: histone H4 acetylation status (positive or negative), white blood count (≤20,000/uL vs. >20,000/uL), cytogenetic risk group, and age (≤35 vs. >35). The latter three variables were chosen since they have been previously identified as prognostic factors in ALL [9-11]. Results were summarized as the odds ratio (OR) or hazard ratio (HR) with 95% confidence intervals (CI). Patients with unknown cytogenetics were not included in the multivariate analysis. The Kaplan-Meier method was used to evaluate histone H4 acetylation and relapse-free and overall survival. P-values were calculated according to the log-rank test.

## Results

### Kasumi Cell Line Results

**Figure 1:**
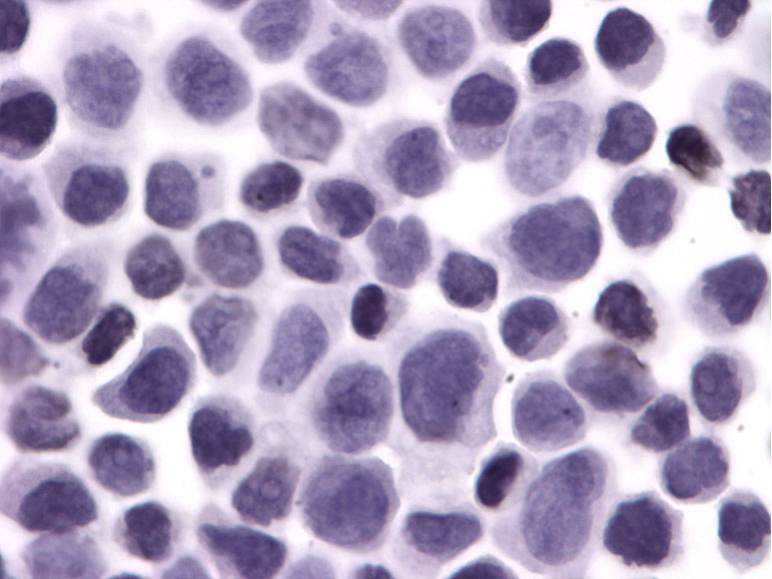
**Histone H4 Acetylation by Immunohistochemistry**. Control sample of Kasumi-1 cells with no sodium butyrate.

**Figure 2:**
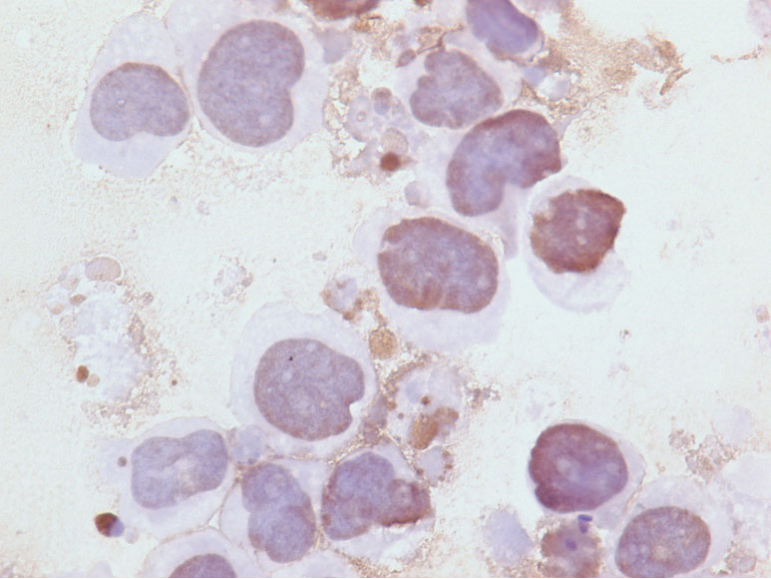
**Histone H4 Acetylation by Immunohistochemistry**. Nuclear staining for acetyl-histone H4 in Kasumi-1 cells with 2 mM of sodium butyrate.

**Figure 3:**
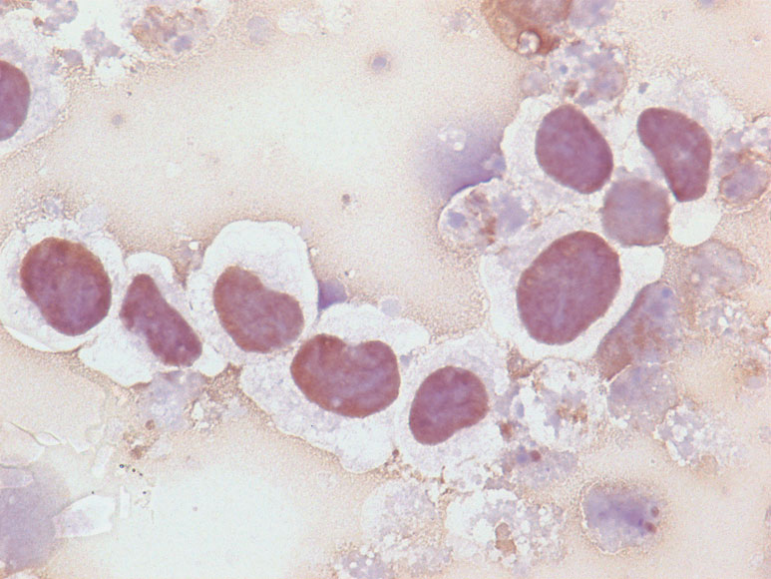
**Histone H4 Acetylation by Immunohistochemistry**. Nuclear staining for acetyl-histone H4 in Kasumi-1 cells with 5 mM of sodium butyrate.

**Figure 4:**
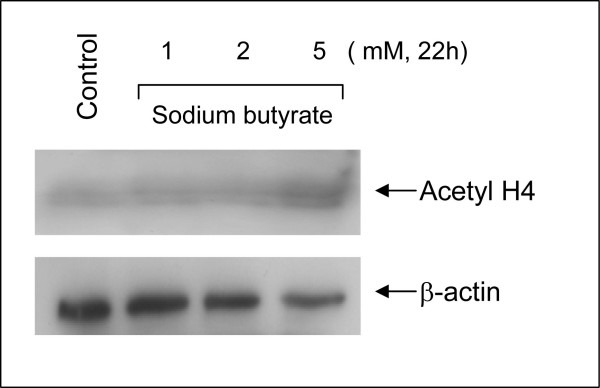
**Histone H4 Acetylation by Western Blotting**. Fresh cultured Kasumi -1 cells were collected and re-suspended in RPMI 1640 medium with 10% fetal bovine serum in the presence or absence of indicated doses of sodium butyrate for 22 hours. Cells were then collected and lysed. Proteins were subjected to SDS-PAGE and immunoblotted with anti-acetyl-H4. The membrane was stripped and reprobed with anti-beta actin antibody. Immunoblotting demonstrated an increasing pattern of histone H4 acetylation with increasing concentrations of sodium butyrate

Untreated Kasumi-1 cells exhibited no staining for acetyl-histone H4. However, upon incubation with the HDAC inhibitor sodium butyrate (2 mM or 5 mM), the Kasumi-1 cells demonstrated increased nuclear staining for acetyl-histone H4 (Figures 1, 2, 3). Western blot analysis of these cells, using the same antibody to acetyl-histone H4 as was used for immunohistochemistry, showed a similar pattern of increasing acetyl-histone H4 levels with higher concentrations of sodium butyrate (Figure 4). This demonstrates the specificity of this antibody for acetyl-histone H4.

### Patient Characteristics

**Figure 5:**
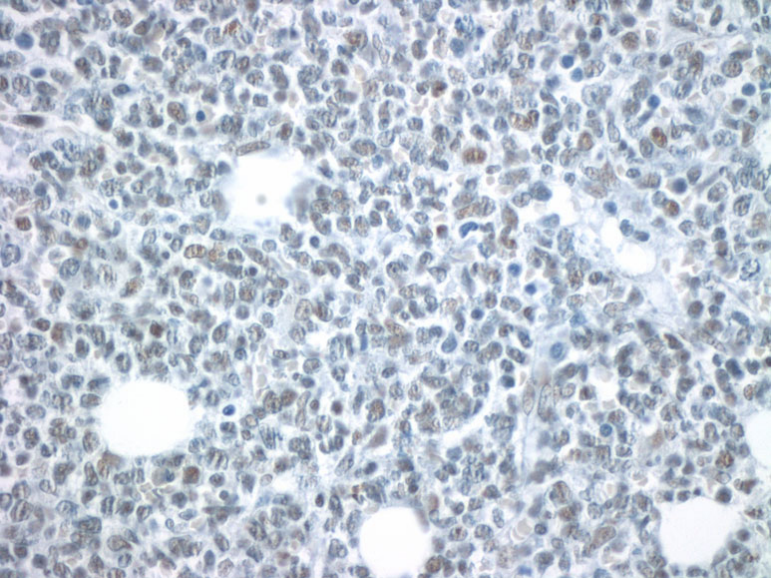
**Histone H4 Acetylation in ALL Patient Samples**. A patient with low levels of histone acetylation (Acetyl-histone H4, original magnification × 400). Olympus BX50 microscope, 100×/1.25 Olympus oil objective, Olympus DP71 camera.

**Figure 6:**
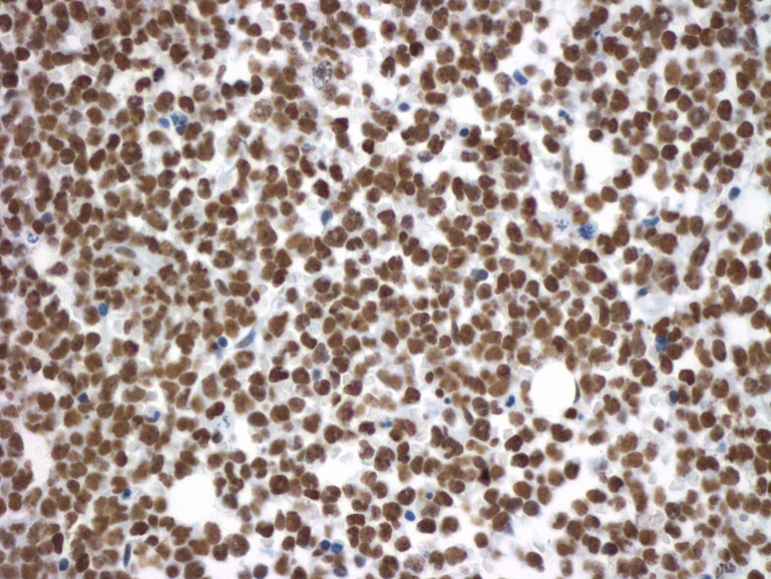
**Histone H4 Acetylation in ALL Patient Samples**. A patient with high levels of histone acetylation (Acetyl-histone H4, original magnification × 400). Olympus BX50 microscope, 100×/1.25 Olympus oil objective, Olympus DP71 camera.

Forty-six patients with ALL treated during this time period had evaluable bone marrow core biopsies. Patient characteristics are described in (Table 1). The median age was 36 years (range 18-66). The median white blood count was 7800/μL (range 840-278,000) and median LDH 763 U/L (range 123-4608). 19 patients (41%) had poor risk cytogenetics, 11 patients (24% ) normal karyotype, 9 patients (20%) miscellaneous abnormalities, and 7 patients (15%) unknown cytogenetics as defined by CALGB criteria. Forty-two patients (91%) had precursor B-cell ALL, 3 patients (7%) T-cell ALL, and 1 patient (2%) mixed lineage leukemia. Thirty-four patients (74%) had strong nuclear expression of acetylated histone H4. Of the T-cell ALL patients, 2 out of 3 patients (67%) had strong nuclear expression of acetylated histone H4. (Figures 5 and 6) illustrate a patient with weak nuclear expression of acetylated histone H4 (Figure 5) and a patient with strong nuclear expression of acetylated histone H4 (Figure 6). There were no associations of age, white blood count at diagnosis, or cytogenetics with histone acetylation.

**Table 1:** Patient characteristics (N = 46 patients)

Patient Characteristics	Value	Range
**Median Age (years)**	36	18-66

**Median white blood count (/μL)**	7,800	840-278,000

**Median LDH (U/L)**	763	123-4608

**Cytogenetic risk groups, n (%)**		
**Poor Risk**	19 (41%)	
**Normal**	11 (24%)	
**Miscellaneous**	9 (20%)	
**Unknown**	7 (15%)	

**Immunophenotype, n (%)**		
**Pre-B**	42 (91%)	
**T-cell**	3 (7%)	
**Mixed Lineage**	1 (2%)	

**Histone Acetylation, n (%)**		
**Present**	34 (74%)	
**Absent**	12 (26%)	

### Treatment

Most patients (38 patients; 82% of patients) received a vincristine/prednisone-based induction as described by the Cancer and Leukemia Group B or the L20 protocol [12-14]. Five patients (11%) received a high dose cytarabine/mitoxantrone regimen [14], and three patients (7%) received double induction (vincristine/prednisone and high dose cytarabine/mitoxantrone) on Southwest Oncology Group protocol S0333. Starting in 2001, patients with Ph + ALL received imatinib mesylate as part of their induction and post-remission therapy. Thirteen patients (28%) proceeded to allogeneic transplant in first remission.

### Response to Therapy and Outcomes

The CR rate after induction therapy was 76%. The median RFS and median OS were both 21.6 months (from the time of diagnosis). Patients who underwent transplantation were not censored at the time of transplant.

### Prognostic Factors on Univariate and Multivariate Analysis

**Figure 7:**
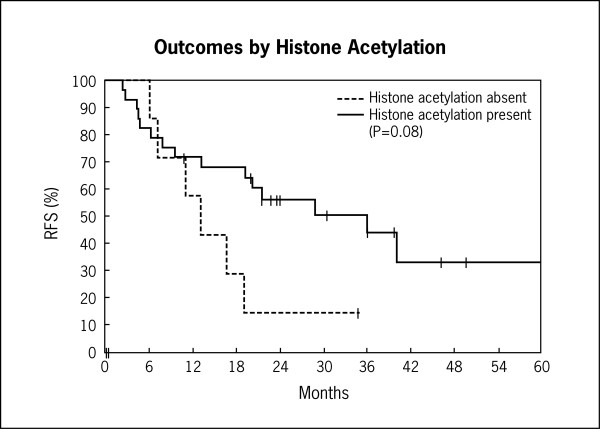
**Kaplan-Meier curves of histone acetylation and relapse-free survival (RFS)**.

**Figure 8:**
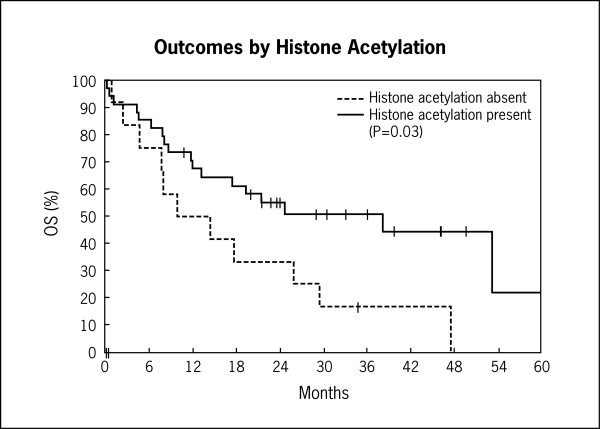
**Kaplan-Meier curves of histone acetylation and overall survival**.

Univariate and multivariate analyses for all patients, including those with poor risk cytogenetics are shown in Tables 2, 3, 4. Kaplan-Meier graphs of histone H4 acetylation and CR, relapse-free survival, and overall survival are shown in Figures 7 and 8. Histone acetylation was associated with an improved OS in all patients (HR 0.45; 95% CI 0.21-0.96, p = 0.038) (Table 4). Age at diagnosis (>35 versus ≤35), white blood count at diagnosis (>20,000/μL versus ≤20,000/μL) and cytogenetic risk group (poor versus others) were associated with a trend towards a decreased overall survival. However, this was not statistically significant (Table 4). On multivariate analysis, histone acetylation was associated with a trend towards a decreased overall survival (when evaluating all CG risk groups) (HR 0.51, 95% CI 0.23-1.13, p = 0.09). However, this was not statistically significant. Treatment (high dose cytarabine/mitoxantrone versus vincristine/prednisone based induction) was not associated with outcome.

**Table 2:** CR Rate: Univariate and Multivariate Analyses for All Patients (N = 46)

Patient Characteristic	Odds Ratio (OR) on Univariate Analysis (95% confidence interval with p-value)	OR on Multivariate Analysis (95% confidence interval with p-value)
**Histone acetylation (Positive versus negative)**	2.24 (0.95-5.28), p = 0.07	2.09 (0.87-5.07), p = 0.10

**Age (>35 years vs. ≤35 years)**	0.67 (0.32-1.39), p = 0.28	0.77 (0.28-2.12), p = 0.61

**White blood count (>20,000/μL versus ≤20,000/μL)**	1.01 (0.47-2.18), p = 0.98	1.18 (0.33-4.26), p = 0.80

**Poor risk cytogenetics versus others**	0.89 (0.41-1.90), p = 0.76	

**Table 3:** Relapse-Free Survival: Univariate and Multivariate Analyses for All Patients (N = 46)

Patient Characteristic	HR on Univariate Analysis (95% confidence interval with p-value)	HR on Multivariate Analysis (95% confidence interval with p-value)
**Histone acetylation (Positive versus negative)**	0.42 (0.16-1.13), p = 0.09	0.55 (0.19-1.59), p = 0.27

**Age (>35 years vs. ≤35 years)**	1.81 (0.75-4.34), p = 0.19	1.39 (0.35-5.72), p =0 .65

**White blood count (>20,000/μL versus ≤20,000/μL)**	3.88 (1.54-9.79), p = 0.004	15.0 (2.41-89.5), p = 0.003

**Poor risk cytogenetics versus others**	2.36 (0.93-6.03), p = 0.07	

**Table 4:** Overall Survival: Univariate and Multivariate Analyses for All Patients (N = 46)

Patient Characteristic	HR on Univariate Analysis (95% confidence interval with p-value)	HR on Multivariate Analysis (95% confidence interval with p-value)
**Histone acetylation (Positive versus negative**	0.45 (0.21-0.96), p = 0.038	0.51 (0.23-1.31), p = 0.09

**Age (>35 years vs. ≤35 years)**	1.81 (0.84-3.91), p = 0.13	2.41 (0.75-7.74), p = 0.14

**White blood count (>20,000/μL versus ≤20,000/μL)**	1.87 (0.87-4.03), = 0.11	3.36 (0.90-12.5), p = 0.07

**Poor risk CG vs. others**	1.95 (0.82-4.68), p = 0.13	

Univariate and multivariate analyses for patients without poor risk cytogenetics are shown in Tables 5, 6, 7. In patients without poor risk cytogenetics (N = 20), there was an impressive association between histone acetylation and CR rate (OR 3.43, 95% CI 1.09-10.8, p = 0.035), RFS (HR 0.13, 95% CI 0.03-0.57, p = 0.008), and OS (HR 0.25, CI 0.09-0.69, p = 0.007) on univariate analysis. This association remained statistically significant in multivariate analysis when age at diagnosis, white blood count at diagnosis, and histone acetylation status were used in the analysis (Tables 5, 6, 7). There was a slight increase in the number of patients with T-cell ALL (10% of total) in the group without poor risk cytogenetics. However, the above results were still valid when evaluating only the B-cell ALL patients.

**Table 5:** CR Rate: Univariate and Multivariate Analysis in Patients without Poor Risk Cytogenetics (N = 20)

Patient Characteristic	Odds Ratio (OR) on Univariate Analysis (95% confidence interval with p-value)	OR on Multivariate Analysis (95% confidence interval with p-value)
**Histone acetylation (Positive versus negative)**	3.43 (1.09-10.8), p = 0.035	3.22 (0.98-10.6), p = 0.053

**Age (>35 years vs. ≤35 years)**	0.61 (0.23-1.58), p = 0.31	0.82 (0.30-2.21), p = 0.82

**White blood count (>20,000/μL versus ≤20,000/μL)**	1.11 (0.32-3.92), p = 0.87	1.06 (0.30-3.79), p = 0.93

**Table 6:** Relapse-Free Survival: Univariate and Multivariate Analysis in Patients without Poor Risk Cytogenetics (N = 20)

Patient Characteristic	HR on Univariate Analysis (95% confidence interval with p-value)	HR on Multivariate Analysis (95% confidence interval with p-value)
**Histone acetylation (Positive versus negative)**	0.13 (0.03-0.57), p = 0.008	0.07 (0.01-0.44), p = 0.005

**Age (>35 years vs. ≤35 years)**	1.95 (0.59-6.50), p = 0.28	1.56 (0.40-6.07), p = 0.53

**White blood count (>20,000/μL versus ≤20,000/μL)**	1.11 (0.32-3.92), p = 0.87	14.9 (2.55-87.6), p = 0.003

**Table 7:** Overall Survival: Univariate and Multivariate Analysis in Patients without Poor Risk Cytogenetics (N = 20)

Patient Characteristic	HR on Univariate Analysis (95% confidence interval with p-value)	HR on Multivariate Analysis (95% confidence interval with p-value)
**Histone acetylation (Positive versus negative**	0.25 (0.09-0.69), p = 0.007	0.24 (0.08-0.68), p = 0.007

**Age (>35 years vs. ≤35 years)**	2.66 (0.93-7.61), p = 0.07	2.49 (0.80-7.79), p = 0.12

**White blood count (>20,000/μL versus ≤20,000/μL)**	3.77 (1.11-12.8), p = 0.033	3.83 (1.06-13.9), p = 0.41

## Discussion/Conclusions

Histone acetylation was associated with an improved CR rate, relapse-free survival, and overall survival in newly diagnosed ALL patients without poor risk cytogenetics. These data suggest that histone H4 acetylation status may help further refine the prognosis of patients with ALL. Such information may be useful in risk-stratifying patients. In the 1980s, Hoelzer et al. defined a prognostic score for ALL patients based on white blood count, age, immunophenotype, and LDH [10]. Over the last decade, cytogenetics have become integral in risk-stratifying patients. Further biological stratification, using techniques such as histone acetylation are likely to become important in the future as we gain a better understanding of the biology of ALL. The methods for defining histone acetylation in this study are straightforward and can be performed routinely in most laboratories, making this approach widely applicable. A more refined prognostic model may be developed evaluating global histone modification patterns, as was done by Seligson et al. in prostate cancer [15]. They evaluated the percent of cells that stained for the histone acetylation and dimethylation of five residues in histones H3 and H4. Although this may lead to a more refined risk score, the methods are more labor intensive. Finally, it is unclear if histone acetylation per se is associated with disease prognosis or if this is a marker for the biological activity of the tumor cells.

Histone acetylation was a prognostic factor on univariate analysis, but not multivariate analysis when including patients with poor risk cytogenetics. Although this may be secondary to inherent differences in the biology (other genetic or epigenetic changes) of the poor risk patients, it is also possible that allogeneic bone marrow transplant abrogated the prognostic impact of histone acetylation in these patients since a majority of patients with poor risk cytogenetics underwent transplant in first remission. There was no significant difference in histone acetylation in patients with poor risk cytogenetics versus other cytogenetic groups.

The limitations of this study are the sample size, heterogeneity of treatment, and the increased number of patients with poor risk cytogenetics. Age, cytogenetics, and white blood count at diagnosis were not significant prognostic factors due to the small sample size. However, there was a trend towards these being prognostic factors. In addition, we took these factors into account in the multivariate analysis. Despite the small sample size, histone acetylation was a significant prognostic factor for patients with normal and miscellaneous cytogenetic abnormalities on both univariate and multivariate analysis. These results should be confirmed in an independent data set with a large number of patients treated on a uniform protocol. Evaluating other histones, such as histone H3, will also be important.

## List Of Abbreviations

(HDAC): Histone deacetylase; (ALL): Acute lymphoblastic leukemia; (CR): Complete remission; (OS): Overall survival; (CG): Cytogenetic; (CALGB): Cancer and Leukemia Group B; (RFS): Relapse-free survival; (OR): Odds ratio; (HR): Hazard ratio; (CI): Confidence interval;

## Competing interests

The authors declare that they have no competing interests.

## Authors' contributions

AA was the principal investigator and takes primary responsibility for the paper. SG, ED, and XZ performed the laboratory work and laboratory analysis for this study. TJ performed the statistical analysis. MK, RS, and MS recruited patients, and MK helped write the paper. EC helped write the paper. SS collected clinical information/data for this study. EH helped with the development of the laboratory assays, review of the laboratory data, and the writing of the paper. The authors report no conflicts of interest.

All authors read and approved the final manuscript.

## Pre-publication history

The pre-publication history for this paper can be accessed here:

http://www.biomedcentral.com/1471-2407/10/387/prepub
